# Baseline iron status and presence of anaemia determine the course of systemic *Salmonella* infection following oral iron supplementation in mice

**DOI:** 10.1016/j.ebiom.2021.103568

**Published:** 2021-09-03

**Authors:** Alexander Hoffmann, David Haschka, Lara Valente de Souza, Piotr Tymoszuk, Markus Seifert, Laura von Raffay, Richard Hilbe, Verena Petzer, Patrizia L Moser, Manfred Nairz, Günter Weiss

**Affiliations:** a Department of Internal Medicine II, Infectious Diseases, Immunology, Rheumatology, Medical University of Innsbruck, Anichstraße 35, Innsbruck A-6020, Austria; b Christian Doppler Laboratory for Iron Metabolism and Anemia Research, Medical University of Innsbruck, Innsbruck A-6020, Austria; c Institute of Pathology, INNPATH, Anichstraße 35, Innsbruck A-6020, Austria

**Keywords:** Iron deficiency anaemia, Salmonella infection, Iron supplementation, Macrophages

## Abstract

**Background:**

Iron deficiency anaemia (IDA) is a major health concern. However, preventive iron supplementation in regions with high burden of infectious diseases resulted in an increase of infection related morbidity and mortality.

**Methods:**

We fed male C57BL/6N mice with either an iron deficient or an iron adequate diet. Next, they received oral iron supplementation or placebo followed by intraperitoneal infection with *Salmonella* Typhimurium (S.Tm).

**Findings:**

We found that mice with IDA had a poorer clinical outcome than mice on an iron adequate diet. Interestingly, iron supplementation of IDA mice resulted in higher bacterial burden in organs and shortened survival. Increased transferrin saturation and non-transferrin bound iron in the circulation together with low expression of ferroportin facilitated the access of the pathogen to iron and promoted bacterial growth. Anaemia, independent of iron supplementation, was correlated with reduced neutrophil counts and cytotoxic T cells. With iron supplementation, anaemia additionally correlated with increased splenic levels of the cytokine IL-10, which is suggestive for a weakened immune control to S.Tm infection.

**Interpretation:**

Supplementing iron to anaemic mice worsens the clinical course of bacterial infection. This can be traced back to increased iron delivery to bacteria along with an impaired anti-microbial immune response. Our findings may have important implications for iron supplementation strategies in areas with high endemic burden of infections, putting those individuals, who potentially profit most from iron supplementation for anaemia, at the highest risk for infections.

**Funding:**

Financial support by the Christian Doppler Laboratory for Iron Metabolism and Anemia Research.

## Graphical abstract

**Figure:**
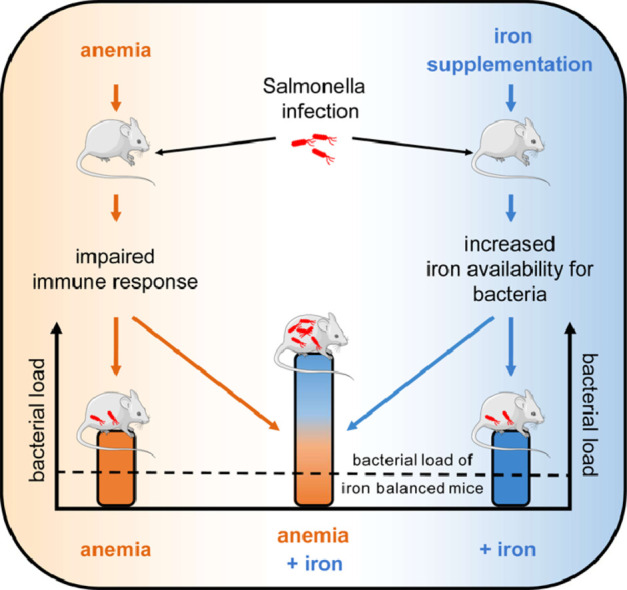

**Research in context**

**Evidence before this study**

Iron deficiency anaemia is the most common form of anaemia worldwide and its prevalence is particularly high in children and pregnant women. Preventive iron supplementation programs in low income countries have been discontinued due to infection-related morbidity and mortality. However, the pathophysiologic relationships and the patient group at greatest risk remain elusive

**Added value of this study**

To gain a better understanding, we mimicked the clinical situation by supplementing anaemic and iron-balanced mice with iron and then infecting them with the Gram-negative bacterium *Salmonella* Typhimurium. Our results showed that the group with the expected greatest benefit, the anaemic mice supplemented with iron, had the highest bacterial load and a reduced survival. Pathophysiologically, we could demonstrate that, on the one hand, iron deficiency anaemia has a negative effect on the immune system and, on the other hand, iron supplementation leads to increased bacterial growth and that these effects are additive

**Implications of all the available evidence**

We were able to show that, surprisingly, the group of patients who would seemingly benefit most from iron administration, is the group who suffer the most from an infection with *Salmonella* Typhimurium.

Alt-text: Unlabelled box

## 1 Introduction

Iron deficiency anaemia (IDA) is the most common form of anaemia worldwide [[1], [2], [3]]. In low-income countries, the prevalence of IDA is high, especially in infants and children, due to insufficient iron intake, increased iron needs during development and/or chronic blood losses. IDA may negatively impact growth, mental and motor development, and cause behavioural problems [4,5]. Iron deficiency can also have negative effects on the development and function of the immune system [6]. Routine iron supplementation is meant to improve development and reduce the prevalence of severe anaemia [7,8]. Despite these positive effects, evidence exists that iron supplementation can result in an increased prevalence of severe infections. A study in 1978 showed that iron-deficient Somali refugees suffered 5 times more often from infections than a placebo group when receiving iron supplements [9]. In 2006, a randomized trial on the island of Pemba was terminated prematurely, as it turned out that routine prophylactic iron supplementation in preschool children increased the risk of infection-related death [10]. The authors speculated that iron supplementation of those children, who are not iron deficient, might be harmful. Although Cochrane reviews [11,12] and a study in Nepal [13] could not fully support those findings, other studies also showed an increased burden of bloody diarrhoea in children with nutritional iron fortification [14]. Although reports and guidelines still support iron supplementation as a first line intervention in children at risk for anaemia, these recommendations have been formulated more cautiously [2,15,16].

The interconnections between dietary treatment of IDA and the incidence of infections are incompletely understood. Also, predictive factors and molecular mechanisms for bacterial infections during oral iron supplementation have not been explicitly defined up to now. To systematically study these issues, we established an animal model that imitates oral iron supplementation in mice that are either iron depleted or have a normal iron availability and studied the course of infection as a function of those variables. *Salmonella* Typhimurium (S.Tm) is a facultative intracellular, Gram-negative pathogen replicating within macrophages and it is highly dependent on iron for achieving full virulence [[17], [18], [19]]. Because of the central role of iron in host-pathogen interaction, immune system driven restriction of iron from invading microbes has been identified as a central anti-microbial defence strategy for which the term nutritional immunity has been coined [[20], [21], [22]]. The idea is to reduce the accessibility of iron for microbes, and those specific processes may differ according to the localization and tissue specific preferences of different pathogens [23,24]. As a consequence, iron availability is also limited for erythropoiesis and results in the development of anaemia of inflammation (AI), which may even aggravate a pre-existing IDA [1,25,26]. In Sub-Saharan Africa, *Salmonella* serovars such as S.Tm cause invasive infections with fatality rates of up to 25% [27,28]. In children, malnutrition, iron deficiency and anaemia are major risk factors for these infections [[29], [30], [31]]. Therefore, we used a mouse model of systemic *S.* Tm infection to study the effects of IDA and dietary iron supplementation on anti-bacterial immunity and disease course.

This study reveals important new knowledge on the multiple effects of anaemia and iron supplementation in an infection model and uncovers an unpredicted group at risk for adverse outcomes in this setting.

## 2 Methods

### 2.1 Mice

**Fig. 1:**
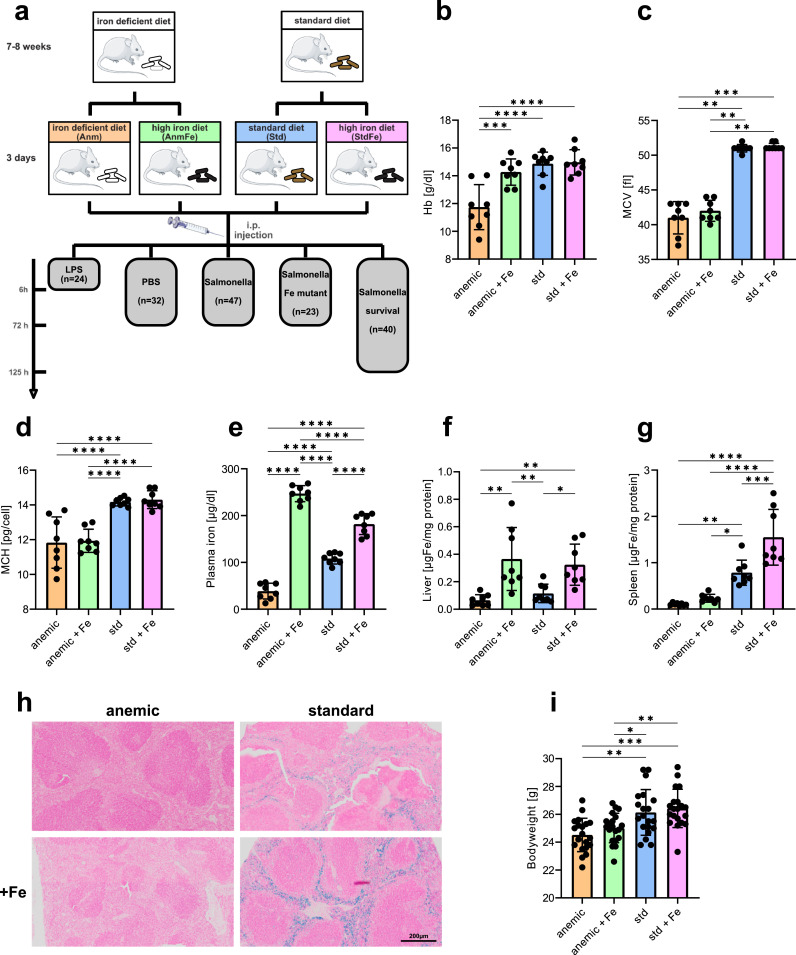
*Iron status of mice.* (a) Scheme of the study setup. Mice received either a low iron diet or a standard iron diet, after six weeks half of each group were changed on a high iron diet to simulate iron supplementation. After additional 3 days mice were infected either with 500 colony forming units (CFU) of *Salmonella* Typhimurium (S.Tm) (*n* = 47), S.Tm for a survival experiment (*n* = 40), lipopolysaccharide (LPS) (*n* = 24), PBS as a control (*n* = 32) or a triple-knockout mutant of S.Tm (*n* = 23). (b–d) Blood scores of uninfected mice. (b) Haemoglobin, (c) mean corpuscular volume (MCV), (d) mean corpuscular haemoglobin (MCH). (e) Plasma iron of PBS treated mice. (f) Non heme tissue iron of the liver and (g) the spleen of PBS treated mice. (h) Representative pictures of Prussian blue stained spleens. Iron appears in blue. (i) Body weight of the mice before infection. Data are presented as mean ± SD. *p < 0.05, **p < 0.01, ***p < 0.001, ****p < 0.0001. Each dot indicates a single mouse. Results are from two pooled identical performed experiments (b-j: *n* = 8, i: *n* = 18–20; per group). One-way ANOVA with Tukey-corrected post-hoc t-tests for multiple comparison was applied for comparison between groups. For non-normal distributed data, a Kruskal-Wallis test was performed. Data are presented as mean ± SD. *p < 0.05, **p < 0.01, ***p < 0.001, ****p < 0.0001. Figure 1a was created using Servier Medical Art templates, which are licensed under a Creative Commons Attribution 3.0 Unported License; https://smart.servier.com (For interpretation of the references to color in this figure legend, the reader is referred to the web version of this article.).

C57BL/6N (Charles River) male mice between 3 and 4 weeks of age were kept under a constant light/dark cycle and had access to food and water *ad libitum*. Half of the mice received a standard diet containing 180 mg iron / kg chow (Ssniff), and the other half a low iron diet containing < 10 mg iron / kg (Altromin) for six weeks. Afterwards, these two groups of mice were again split into two subgroups, whereas one part was switched to a high iron diet containing 25 g iron / kg (Altromin) and the other part remained on the initial diet. Three days later mice received a intraperitoneal (i.p.) injection of either *Salmonella enterica subsp. enterica serovar Typhimurium ATCC 14028* (S.Tm), PBS or LPS (Fig. 1a).

### 2.2 Induction of systemic infection with *Salmonella* Typhimurium in mice

Differentially fed mice were infected with S.Tm or with a triple mutant strain of *Salmonella* with deletion of two major iron uptake systems (∆entC ∆feo) [32,33] and the lately described Mn^2+^ but also Fe^2+^ transporter ∆sitABCD for 72 h (Fig. 1a) [34]. For systemic infection, bacteria were grown under sterile conditions in LB broth (Sigma) to late-logarithmic phase, counted with the CASY cell counter (Schärfe System), and mice were then infected i.p. with 500 colony forming units (CFU), proofed by plating of an aliquot, of the indicated *Salmonella* strain in 200 µl of PBS. Control mice received 200 µl of PBS without bacteria.

The bacterial load of organs was determined by plating serial dilutions of organ homogenates on LB agar (Sigma) under sterile conditions and the number of bacteria calculated per gram of tissue.

When indicated, 2 mg/kg bodyweight of lipopolysaccharide (LPS) from *Salmonella* Typhimurium (Sigma) in 200 µl PBS were i.p. injected and mice were terminated after 6 h.

For each *in vivo* experiment, surface body temperature was measured in at least 8 h intervals. Loss of reflexes (righting and grabbing reflex) and/or body temperature (surface) drop of the animal of more than 4 °C compared with the pre-infection baseline and/or loss of bodyweight < 20% were assumed a humane endpoint for infection and survival experiments.

### 2.3 Ethics

All animal experiments were performed in accordance with the Austrian Experimental Welfare Act 2012 (Tierversuchsgesetz 2012) and were approved by the Federal Ministry of Science and Education (approval no. BMWFW-66.011/0096-V/3b/2018 and BMWFW-66.011/0063-WF/V/3b/2017). All experiments were performed with the aim to keep animal pain, stress and numbers to a minimum as stated in our animal approval.

### 2.4 Blood and plasma measurements

Blood counts were performed using a scil VET abc (Lab Technonolgies) blood counter. For further analysis, heparin blood was centrifuged twice to obtain plasma. 50 µl of plasma were used for measurement of plasma iron using the QuantiChrome^TM^ Iron Assay (BioAssay Systems) following the manufacturer's protocol. For the detection of enhanced labile plasma iron (eLPI) concentrations in plasma we used a FeROS eLPI kit (Afferix) which was performed according to the manufacturer's protocol and described in detail elsewhere [35]. eLPI concentrations are presented in arbitrary unites [AU].

ELISAs were used to measure hepcidin (HMC-001 Hepcidin Murine-Compete ELISA Kit Intrinsic LifeSciences (ILS)) and transferrin (Tfn) (Mouse Tfn ELISA Kit, Abcam) levels in mouse plasma. For the measurement of cytokines in the plasma, a Mouse ProcartaPlex Mix&Match 8-plex (ThermoFischer Sientific) was performed according to the manufacturer's protocol.

Transferrin (Tfn) saturation (TSAT) was calculated based on plasma iron and Tfn measurement using the following formula:

TSAT [%] = (Fe [µg/dL]/Tfn [mg/dL]) × 70.9

### 2.5 Tissue iron measurement

The determination of non heme tissue iron content was conducted with acid-hydrolysed tissue homogenates with a colorimetric method employing bathophenanthroline disulfonic acid and L-ascorbic acid in a sodium acetate assay buffer as described elsewhere [36]. The calculated iron quantity was normalized to protein concentrations assessed by the Bradford method, for each sample.

### 2.6 Histopathology

Histologic examination of spleens and livers was performed on formalin-fixed tissue sections stained with Prussian blue according to standard protocols. For image acquisition, an Olympus BX61VS slide-scanner equipped with a 20x objective with a numerical aperture of 0.75, and the OlyVIA software were used.

### 2.7 Bacterial growth assay

IMDM media (Thermo Fischer) was supplemented with 20% of plasma from uninfected mice receiving the different diets. To each sample 4 × 10^6^ /ml CFU of S.Tm were added and incubated on a shaker for 4h at 37 °C. The number of bacteria was determined with the CASY system (Schärfe System).

### 2.8 RNA preparation from tissue, reverse transcription and TaqMan real-time PCR

This was carried out as described previously [37]. In brief, total RNA was prepared from liquid nitrogen-frozen mouse tissues using acid guanidinium thiocyanate-phenol-chloroform extraction with peqGOLD Tri-Fast™ (Peqlab). For reverse transcription 2 µg RNA, random hexamer primers (200 ng/µl) (Roche), dNTPs (10 mM) (GE Healthcare LifeSciences) 20 U RNasin (Promega) and 200 U M-MLV reverse transcriptase (Invitrogen) in first strand buffer (Invitrogen) were used. TaqMan real-time PCR was performed on a CFX96 light cycler (Bio-Rad). Ssofast Probes Supermix and Ssofast EvaGreen Supermix (Bio-Rad Laboratories GmbH) were used according to the manufacturer's instructions. Real-time PCR reactions were performed on a CFX Cycler and analyzed with CFX software (BioRad). Gene expression was calculated using the ΔΔct method using *Rpl4* for murine samples and *16sRNA* for bacterial samples as reference transcripts.

The following TaqMan PCR primers and probes were used (primer forward; primer reverse; probe):

Mouse:

*IL-6*: 5’-TGTTCTCTGGGAAATCGTGGA-3’; 5’-AAGTGCATCATCGTTG TTCATACA-3’; FAM,5’-ATGAGAAAAGAGTTGTGCAATGGCAATTCT G-3’, BHQ1 *IL-1b*: 5´-GATGAGGACATGAGCACCTTCTT-3´; 5´-GCAGG TTATCATCATCATCCCA-3´; FAM,5´-CATCTTTGAAGAAGAGCCCATCCT CTGTGA-3´,BHQ-1 *Tnfa*: 5’-TTCTATGGCCCAGACCCTCA-3’; 5’-TTGC TACGACGTGGGCTACA-5’; FAM,5’-CTCAGATCATCTTCTCAAAATTCGAGTGACAAG-3’,BHQ 1*IL-10*: 5’-CCAGAGCCACATGCTCCTAGA-3’; 5’-TGGTCCTTTGTTTGAAAGAAAGTCT-3’; FAM,5’-TGCGGACTGCCT TCAGCCAGG-3’,BHQ-1 *Rpl4*: 5’-CGCTGGTGGTGGAAGATAAGG-3’; 5’-CGGTTTCTCATTTTGCCCTTG-3’; Cy5,5’-CAGCCTCCTTGGTCTTCT TGTAACCTTC-3’,BHQ2

Salmonella:

*16S rRNA*: 5´-GAAATGCGTAGAGATCTGGAGG-3´; 5´-CACAACCTCCAAGTAGACATCG-3’; Cy5, 5’-TAATCCTGTTTGCTCCCCACGCT-3’,BHQ2 *fur*: 5’-ATGGGTGAAGAAATCGGTCTGG-3’; 5’-GATGGTCGTGATGATGCTGTTG-3’ *invF*: 5’-GCAGGATTAGTGGACACGAC-3’; 5’-TTTACGATCTTGCCAAATAGCG-3’ *sicA*: 5’-ATTTGGGATGCCGTTAGTGAAG-3’; 5’-TAAACCGTCCATATCTTGAGG-3’; FAM,5’-ATCCCATGAACGTCTTTTAGCGTGGC-3’

BHQ1 *sitB*:

5’-CTGCGATTACCGTTGATCAAGTC-3’; 5’-CCGGAACCATTTACGCCTACC-3’; *sodB*: 5’- TCGTTTCTACCTCAAATGCCG3’; 5’-GCCCAGAAATGCTCCAGATAG-3’; FAM,5’-TCCCACACATCAACCGTCAGC AA-3’,BHQ1 *ssaG*: 5’-TGTAAGGCAAATTGCGCTTTAA-3’; 5’-AGGCCATTAATGACAAAATGAATG-3’ *sseA*: 5’-CAAATCCGGGCTAAGGTGAGT-3’; 5’-CTTCTCGGCCTCCTGGTTAAC-3’

### 2.9 Peritoneal associated macrophages (PAM)

The peritoneum of mice was washed twice with 10ml of pre-warmed (37 °C) PBS which was gently injected using a 23 g needle without harming inner organs. PBS was aspirated from the peritoneum and centrifuged at 300 g for 5 min. Samples of 5 mice per group were pooled to obtain enough protein for the Western blot analysis as described [37].

### 2.10 Flow cytometry analysis

All data were acquired on a CytoFLEX S (Beckman Coulter) and analysed with FlowJo 10.7.0 software (Beckton Dickinson). Spleen cells were isolated by mashing the organ through a 100µm cell strainer (Corning) with PBS. Flow cytometry staining was performed as described previously [36,38] with the following antibodies: anti-CD3e (PE-eFluor 610, 145-2c11 Thermo Fisher Scientific Cat# 61-0031-82, RRID:AB_2574514), anti-CD45 (PE-Cy5, 30-F11, BD Biosciences Cat# 553082, RRID:AB_394612), anti-Ly-6G (PerCP-eFluor710, 1A8-Ly6g, Thermo Fisher Scientific Cat# 46-9668-80, RRID:AB_2573892), anti-CD19 (Biotin, 6D5, BioLegend Cat# 115503, RRID:AB_313638) and Streptavidin (BV605, BD Horizon Cat# BDB563260).

For staining of T cell subsets splenic cells were isolated by mashing the spleen through a 100µm strainer followed by a re-stimulation for 3,5 h in 1 ml of RPMI 1640 (PAN BIOTECH) supplemented with 10% FBS (PAN BIOTECH), 1% PEN – STREP (Lonza), 1% L-Glutamine (Lonza) 10 µg/ml Brefeldin A (Sigma), 100 ng/ml Ionomycin (Sigma) and 50 ng/ml PdBu (Sigma). After re-stimulation, surface proteins were stained for 10 min at 4 °C, washed once with PBS, and centrifuged for 5 min at 300 g. For permeabilization, the cell pellet was resuspended in FixPerm (BD Biosciences) and incubated for 20 min at 4 °C followed by a washing step with PermWash (BD Biosciences). After another centrifugation step, the pellet was resuspended in 100 µl Triton Buffer (Carl Roth) and incubated for 10 min on ice. The cells were washed again with PermWash and incubated with the cytokine-antibody mix for 45 min at room temperature. After another wash step with PermWash the cells were resuspended in PBS and were ready for analysis. The following antibodies were used: for surface staining anti-CD4 (PerCP/Cyanine 5.5, GK1.5, Bio Legend Cat# 100434), anti-CD8a (Alexa Fluor 700, 53-6.7, BD Biosciences Cat# 557959, RRID:AB_396959), anti-CD45 (BV510, 30-F11, BD Biosiences Cat# BDB563891), anti-CD3 (PE–Cy 5, 17A2, BD Biosciences Cat# 555276) and for intracellular staining anti-TNFa (APC, MP6-XT22, eBiosciences Cat# 17-7321-82), anti-IFNg (PE, XMG1.2, BD Pharmingen Cat# 554412), anti-IL-4 (BV421, 11B11, BD Biosciences Cat# 562915, RRID:AB_2737889) and anti-IL-17A (APC-Cy7, TC11-18H10, BD Pharmingen Cat# 560821).

### 2.11 Western blot

Protein extraction and Western blotting were performed as described [37]. The following antibodies were used: a rabbit FPN1 antibody (1:2000; Eurogentec, NRU 451443), a mouse TFR antibody (1:1000; Sigma Cat# SAB4300398), a rabbit ferritin antibody (1:500; Sigma), a rabbit actin antibody (1:500; Sigma Cat# A2066), and appropriate HRP-conjugated secondary antibodies (1:2000, anti rabbit; Dako Cat# P0399 1:4000, anti mouse; Dako Cat# P0447). For quantification, densitometry data were acquired on a ChemiDoc Touch Imaging System (Bio-Rad) and analyzed with Image Lab 5.2.1. (Bio-Rad).

### 2.12 Statistical analysis

All data were analysed at the end of the experiments except for bodyweight, which was measured right before injection of bacteria, LPS or PBS. Statistical analysis was carried out using GraphPad Prism version 8 for Windows (GraphPad Software). Data are presented as mean ± SD unless otherwise specified. Significant differences between groups were determined using a one-way ANOVA or two-way ANOVA (more than 2 groups, two factors) with Tukey-corrected post-hoc tests for multiple comparison. Data were tested for normality using the Shapiro Wilk test. For non-normal distributed data, a Kruskal-Wallis test was performed with Dunn's post hoc test. For the correlation of CFU and TSAT a nonlinear regression model polynomial (quadratic) was used. For this model outlier were excluded when Q < 0.001%. To compare survival curves we used a Gehan-Breslow-Wilcoxontest with Holm-Šídák post-hoc correction for multiple comparisons. For all other results outliers were calculated using the ROUT test (Q = 1%) and excluded outliers are mentioned in the figure legends. In five experiments a total of 166 mice were used and all animals were analysed and included in the results. The sample size for each animal experiment was estimated based on previous experience, usually with 5-7 animals for bacterial infections and LPS treatment respectively and 3-5 animals for control groups. At the date of delivery, mice (age 3-4 weeks) were randomly assigned to the different diet groups as well as infection or control groups. The lab members who were responsible for setting up the diets and infecting the mice were different from those terminating the animals and collecting the data at the end of the experiments. p < 0.05 was used as the significance threshold.

### 2.13 Role of the funding source

The funders had no role in study design, data collection, analysis and interpretation, writing and submission of the manuscript. All authors had complete access to the data.

## 3 Results

### 3.1 Iron status of mice

To mimic iron supplementation in regions with high prevalence of IDA, we used 2 types of chow which varied in their iron content. Using different dietary protocols we were able to study four groups of mice, namely iron deficient anaemic mice (Anm), iron deficient anaemic mice receiving iron supplementation (AnmFe), iron balanced mice (Std) and iron balanced mice with iron supplementation (StdFe) (Fig. 1a). Uninfected Anm mice had a microcytic, hypochromic anaemia with low hemoglobin (Hb), low mean corpuscular volume (MCV) and low mean corpuscular hemoglobin (MCH), a combination typical of IDA (Fig.1b–d). Upon iron supplementation, the Hb level in these mice rose almost to the level of the Std group, although MCV and MCH remained low. In contrast, iron supplementation in Std mice had no effect on Hb, MCV or MCH (Fig. 1b–d). When studying body iron distribution in the four groups we found that the iron content in plasma increased more than six-fold in Anm mice as compared to 1.6-fold in Std mice upon iron supplementation (Fig. 1e). This points to a more efficient iron absorption in anaemic mice, promoted as well by low hepcidin levels (Fig. 3f), which is in line with the known metabolic adaptions of iron homeostasis genes upon anaemia [1,39]. Of relevance, iron supplementation increased tissue iron in the liver of AnmFe and StdFe groups to the same level, whereas in the spleen tissue iron content only increased in the StdFe group, and no obvious increase was found in the AnmFe group (Fig. 1f–g). Prussian blue staining of the spleen confirmed the results of the tissue iron measurements and revealed iron accumulation in macrophages in the Std and, more pronounced, in the StdFe group (Fig. 1h). Prior to infection, the bodyweight of Anm and AnmFe mice was significant lower compared to Std and StdFe mice, which most likely reflects the growth and development retardation caused by long-term iron deficiency (Fig. 1i).

Taken together, our animal model with different iron status and iron distribution showed to be a competent model to mimic a situation of iron supplementation to children with underlying IDA or balanced iron status and allows us to study its impact on the course of bacterial infection.

### 3.2 Iron supplementation to anaemic mice is associated with a detrimental course of infection with *Salmonella* Typhimurium

**Fig. 2:**
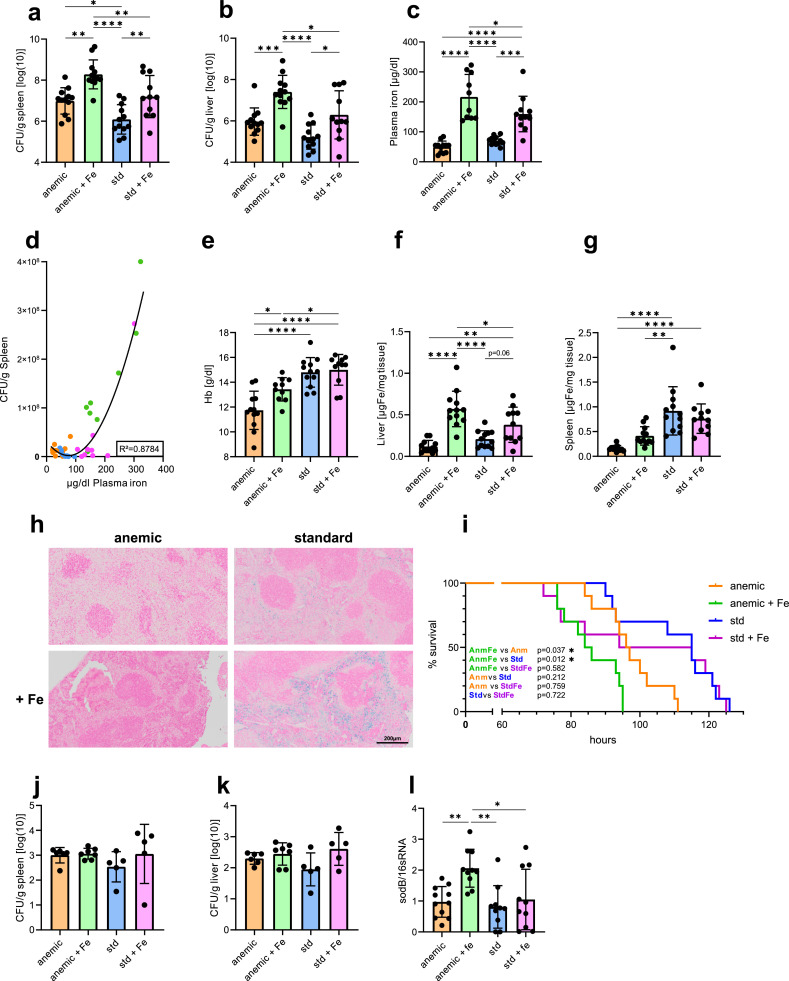
Iron supplementation to anaemic mice is associated with a detrimental course of infection with *Salmonella* Typhimurium. (a) Bacterial load of the spleens (b) and livers of differentially fed mice. Bacterial load was determined by plating serial dilutions of tissue lysates and are shown in log10. (c) Plasma iron of mice infected with *Salmonella* Typhimurium (S.Tm). (d) Regression analysis of CFU of spleens and the amount of plasma iron. A second or polynomial (quadratic) regression was performed with outliers set at *Q* = 0.001%. R squared is mentioned in the graph. One outlier was detected for the group of Anm mice, and two outliers for each the AnmFe and StdFe groups. (e) Haemoglobin of infected mice. (f) Non heme tissue iron of the liver and (g) the spleen of S.Tm infected mice. (h) Representative pictures of Prussian blue stained spleens of S.Tm infected mice. Iron appears in blue. (i) Kaplan-Meyer curve displays mouse survival of the different groups over time. (j-k) Differentially fed mice were infected with 500 CFU of a triple mutant strain of S.Tm (*∆entC ∆sitABCD ∆feo*). Bacterial load of (j) the spleen and (k) the liver are shown in log10. (l) qPCR results of bacterial *sodB* gene-expression. One-way ANOVA with Tukey-corrected post-hoc t-tests for multiple comparison was applied for comparison between groups. Data are presented as mean ± SD. *p < 0.05, **p < 0.01, ***p < 0.001, ****p < 0.0001. Each dot indicates a single mouse. Results of (a–g) and (l) are from two identical performed experiments (*n* = 10–12). Results from (i) is from a single experiment with *n* = 10 for each group Results of (j,k) are from a single experiment (*n* = 5–7). One-way ANOVA with Tukey-corrected post-hoc t-tests for multiple comparison was applied for comparison between groups. Two-way ANOVA with Tukey-corrected post-hoc t-tests for multiple comparison was performed for the comparison of the different groups of infected and PBS treated mice. To compare survival curves a Gehan-Breslow-Wilcoxontest with Holm-Šídák post-hoc correction for multiple comparisons was used. Data are presented as mean ± SD. *p < 0.05, **p < 0.01, ***p < 0.001, ****p < 0.0001 (For interpretation of the references to color in this figure legend, the reader is referred to the web version of this article.).

To study the effects of baseline iron status and oral iron supplementation on the course of bacterial infection, mice were subjected to an intraperitoneal (i.p.) injection with 500 colony-forming units (CFU) of the Gram-negative, intracellular bacterium *Salmonella* Typhimurium (S.Tm) or PBS as a control (Fig. 1a). After 72 h of infection, we observed significantly higher bacterial loads in spleens and livers of iron-supplemented mice, and bacterial loads of AnmFe were significantly higher compared to all other groups (Fig. 2a-b). Interestingly, the bacterial load of Anm mice was higher than in Std mice and even comparable to iron supplemented Std mice (StdFe). Plasma iron levels were highest in AnmFe followed by StdFe mice, whereas Anm mice presented with the lowest plasma iron concentrations at 72 h after infection (Fig. 2c). Next, we analysed the association of plasma iron levels with bacterial load of the spleen in the entire study collective. Intriguingly, we found a significant U-shaped correlation between plasma iron concentrations and bacterial burden, meaning that low and especially high levels of plasma iron were associated with increased bacterial loads in the spleen (Fig. 2d). In regard to blood scores, infected mice did not show changes in haematocrit in comparison to uninfected mice (Suppl. Fig. 1a). Respectively, Hb levels were similar in infected and PBS treated mice except for the AnmFe group, which did not reach levels of the uninfected group (Figs. 2e, 1b). Of interest, while liver iron concentration of infected mice largely paralleled the findings seen with plasma iron in the different groups, splenic tissue iron concentrations were lower in infected anaemic than non-anaemic mice, and iron supplementation had no significant effect on splenic iron concentrations (Fig. 2c,f,g). As spleens of infected mice increase in size (splenomegaly) we performed in addition a calculation of absolute splenic tissue iron content. Herein we found higher splenic iron content in the Std groups, and iron supplementation increased iron levels, although to a lesser extent in infected AnmFe mice (Suppl. Fig. 1b). Prussian blue stained splenic histological slides of infected mice fit to the data of absolute splenic iron (Fig. 2h, Suppl. Fig. 1b) and were comparable to those from uninfected mice (Fig. 1h), with more prominent iron staining in mice on the standard iron diet (Std, StdFe). To investigate whether changes in bacterial loads translate into different outcomes, we monitored survival of infected mice. Thereby, AnmFe mice had the worst outcome and succumbed to death earlier than the Anm and Std groups. Of note, both groups with iron supplementation had an onset of mortality between 70 to 80 h of infection. However, StdFe mice then stabilized for about 30 h whereas AnmFe did not. Onset of death in Anm mice was later but then almost paralleled the mortality kinetics of AnmFe mice (Fig 2h). To verify if the observed differences in bacterial loads are limited to bacterial iron acquisition, we infected mice on the different iron diets for 72 h with a triple mutant strain of *S.Tm,* lacking two important iron acquisition mechanisms (*∆entC, ∆feo*) [32] and also lacking one lately described Mn^2+^ and Fe^2+^ transporter (*∆sitABCD)(34)*. After three days of infection, the bacterial numbers were not significantly different between the four groups in liver and spleen (Fig. 2j-k). Specifically, the increased bacterial load in the AnmFe group observed after infection of mice with wild-type S.Tm became no longer evident in liver and spleen when using the mutant bacterial strain. This highlights the high importance of accessible iron for bacterial growth, maintenance and/or virulence. In a next step, we addressed the question whether the high plasma iron levels detected in AnmFe infected mice also correlated with increased accessibility or availability of iron for S.Tm. Therefore, we studied the expression of *sodB*, a bacterial superoxide dismutase. *sodB* is neither influenced by pH nor the presence of reactive oxygen species but is affected by intracellular iron levels, as it is indirectly regulated by the central iron regulator *fur* [41]. In this setup, the bacteria in spleens of AnmFe mice had significantly higher levels of *sodB* compared to the other groups (Fig. 2l). This might indicate that the bacteria within the spleens of mice of the AnmFe group, could have access to more intracellular iron than in any other group. We then studied whether or not altered iron availability for bacteria could affect the expression of bacterial pathogenicity factors. However, analysis of the expressions of selected genes of the Salmonella pathogenicity island gene cluster (SPI) 1 (*invF, sicA*), which is involved in epithelia cell invasion, and SPI 2 (*ssaG, sseA*), which is necessary for intracellular survival and replication, showed some iron dependent changes in certain genes but no significant differences between the four groups (Suppl. Fig. 1c–f). When studying the expression of the central iron regulator *fur* and one of its target genes *sitB*, which is part of the *sitABCD* operon, no significant differences were found (Suppl. Fig. 1 g-h). Nevertheless, the sitABCD operon is also regulated by MntR in response to Mn2+, which complicates the interpretation.

These data obtained so far, suggested that iron supplementation leads to a worse outcome of infection on the basis of a pre-existing IDA, which is linked to increased iron access for bacteria.

### 3.3 Regulation of iron dependent genes and proteins

As iron availability for bacteria appears to play a role for the outcomes of the observed phenotypes, we next evaluated the expression pattern of the host iron dependent genes and proteins.

**Fig. 3:**
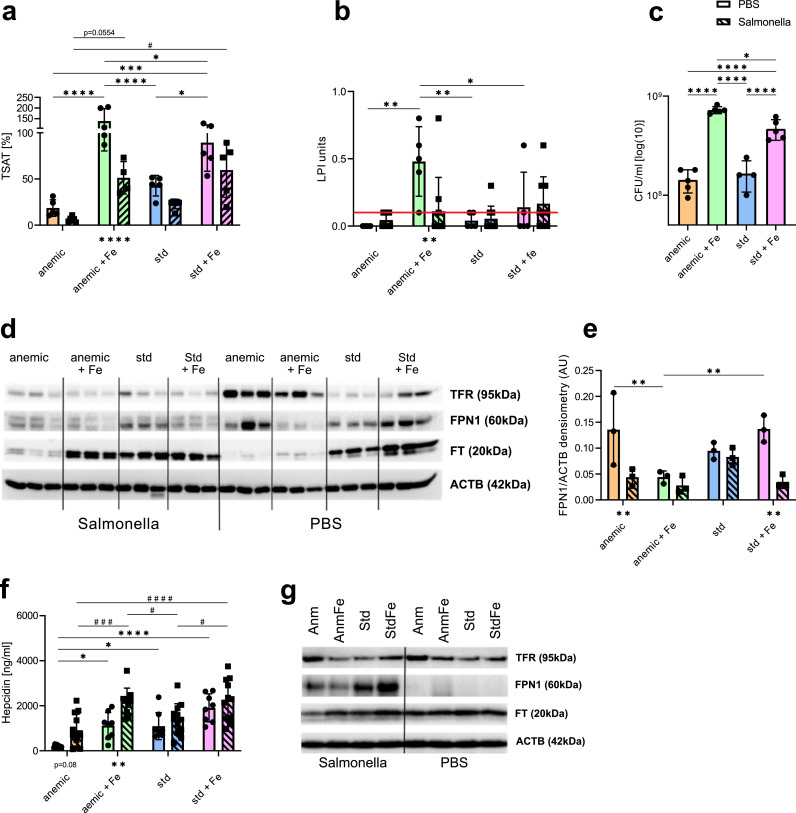
Regulation of iron dependent genes and proteins. (a) Transferrin saturation (TSAT) of *Salmonella* Typhimurium (S.Tm) infected and PBS treated mice receiving the different iron diets. Transferrin saturation was calculated (non heme plasma iron /plasma transferrin) * 70.9. (b) eLPI (enhanced labil plasma iron) measurement of infected and PBS treated mice, which is indicative for non-transferrin bound iron (NTBI). According to the manufactures protocol samples above the threshold (indicated by the red line (≥ 0.2)) are positive. (c) Bacterial growth assay. Bacteria grew for 4h in iron free IMDM media supplemented with 20% of the plasma of uninfected mice receiving the different iron diets. Data is shown in CFU/ml. (d) Western Blot of transferrin receptor (TFR), ferroportin (FPN1), ferritin (FT) and actin (ACTB) as a reference protein. Triplicates of differently fed and either S.Tm infected or PBS treated mice. (e) Quantification of FPN1 from the western blot results. (f) Hepcidin Elisa from plasma of infected and uninfected mice receiving the different diets. (g) Western blot of peritoneal associated macrophages (PAM) from infected and PBS treated mice receiving the different diets. Transferrin receptor (TFR), ferroportin (FPN1), ferritin (FT) and actin (ACTB) as a reference protein. Each dot/square indicates a single mouse. Results of (a) and (f) are from two identical performed experiments (PBS: *n* = 8, S.Tm: *n* = 10–12). Results of (b,c) and (g) are from a single experiment (PBS: *n* = 5, S.Tm: *n* = 4–6). One-way ANOVA with Tukey-corrected post-hoc t-tests for multiple comparison was applied for comparison between groups. Two-way ANOVA with Tukey-corrected post-hoc t-tests for multiple comparison was performed for the comparison of the different groups of infected and PBS treated mice. S.Tm infected mice are shown in bars with a diagonal line pattern and black squares for each mouse and PBS treated mice in blank bars with black circles for each mouse. Differences between infected and PBS treated are shown underneath the bars. When S.Tm and PBS treated mice are shown on the same graph significances between the S.Tm infected mice are shown using # and significances between PBS treated groups are shown using *. Data are presented as mean ± SD. *p < 0.05, **p < 0.01, ***p < 0.001, ****p < 0.0001, #*p* < 0.05, ###*p* < 0.001, ####*p* < 0.0001.

Transferrin (Tfn) is the most important iron carrier in the blood, whose circulating concentrations decrease during inflammation in order to reduce iron access for certain microbes [42,43]. Surprisingly, plasma Tfn levels increased in the two anaemic groups upon infection as compared to uninfected animals, whereas it remained largely unchanged in the Std and StdFe group upon infection (Suppl. Fig. 1i). Deduced from these results and the plasma iron content, we calculated the transferrin saturation (TSAT). In PBS treated AnmFe mice, the concentration of iron in the plasma exceeds the binding capacity of Tfn, resulting in a calculated TSAT of 138% (AnmFe) and 89% (StdFe), whereas the Anm mice had 18% and the Std mice 42% (Fig. 3a). With TSAT levels exceeding 80%, non-transferrin-bound iron (NTBI) can occur in the plasma [44,45]. A measurement of the enhanced labile plasma iron (eLPI), which is indicative of total NTBI, confirmed a higher amount for the AnmFe mice in comparison to StdFe mice without infection (Fig. 3b). The fact that infected AnmFe mice had a higher non-heme tissue iron content in the liver support these findings, as the liver is one of the first organs to take up NTBI [46] (Fig 2f). Infection resulted in a significant reduction of TSAT levels in all groups but TSAT levels remained higher in iron supplemented mice (AnmFe, StdFe) as compared to those animals remaining on their previous diet (Fig. 3a). These data suggest that at the time point of infection, iron supplemented mice (AnmFe and StdFe), independent of their basal iron status, offer bacteria an iron rich environment. To further investigate the influence of plasma iron (transferrin bound iron and NTBI) on bacterial growth, we performed an *in vitro* bacterial growth experiment using iron free IMDM media supplemented with 20% of plasma from uninfected mice receiving the different diets. After 4 h of growth, we found that with higher baseline plasma iron levels and increased TSAT, bacterial growth was strongly promoted *in vitro*. Therefore, the most sustained growth promoting effect was observed with plasma obtained from AnmFe and Std Fe mice (Fig. 3c). These data nicely fit to plasma iron levels in infection (Fig. 2c), which were highest in AnmFe and StdFe mice, suggesting that increased circulating iron levels can directly promote bacterial growth. As *Salmonella* Typhimurium is an intracellular bacterium residing mainly in macrophages, we next evaluated the expression of key iron proteins in the spleen, because of its high density of macrophages. In uninfected animals, we found low levels of the iron storage protein ferritin (FT) in Anm and AnmFe mice, which was paralleled by increased transferrin receptor 1 (TFR) expression as compared to Std. conditions, both being indicative for iron deficiency (Fig. 3d, Suppl. Fig. 1j-k). The finding that iron supplementation did not increase ferritin levels of uninfected anaemic mice within three days may be related to the fact that iron was primarily used for erythropoiesis as reflected by increased hemoglobin levels (Figs. 1b, 3d, Suppl. Fig. 1k). Of note, while the expression of the sole known iron exporter ferroportin (FPN1) was high in uninfected Anm mice, it decreased upon iron supplementation (Fig. 3d-e). Std and StdFe mice without infection presented with low TFR, besides detectable ferritin and FPN1 levels. In addition there was a tendency towards higher expression of the latter two proteins upon iron supplementation (Figs. 3d-e, Suppl. Fig. 1j-k). Upon infection, TFR expression was found to be low in all groups (Figs. 3d, Suppl. Fig. 1j). In contrast, while FT levels increased in AnmFe and decreased in StdFe mice compared to uninfected ones, FT levels in Std and Anm mice remained largely unchanged upon infection, with Anm being lowest in both treatments (Figs. 3d, Suppl. Fig. 1k).

FPN1 has been described to be decisive during infection with intracellular pathogens as it can determine iron availability for the microbes [[47], [48], [49]]. After 72 h of infection, FPN1 expression was low in all groups except for Std mice (Fig. 3d,e), indicating that both iron supplementation and anaemia contribute to reduce splenic FPN1 expression during infection, a situation which would favour the growth of intracellular pathogens.

Since FPN1 expression is mainly regulated post-translationally upon interaction with hepcidin, we studied protein levels thereof. In agreement with the known regulation of hepcidin by iron availability and anaemia [50], hepcidin levels were the lowest in uninfected anaemic mice and increased upon iron supplementation (Fig. 3f). Upon infection, hepcidin plasma levels were increased after 72 h, and the highest concentrations were observed in mice supplemented with iron (AnmFe, StdFe). This is accordance with the observed alterations of FPN1 expression in spleens of infected mice, being the lowest in the AnmFe and StdFe groups (Fig. 3d–f).

To study iron homeostasis specifically in macrophages, which are the major habitat for *Salmonella* in this type of infection, we isolated peritoneum associated macrophages (PAM) from all groups of mice for further analysis. Interestingly, in non-infected mice TFR expression was higher in Anm mice as compared to all other groups, whereas ferritin and FPN1 protein expression were not different among all groups (Fig.3g). In contrast to the data from whole spleen lysates, FPN1 protein levels were low in uninfected mice but significantly increased following infection, with the highest expression seen in Std and StdFe mice. Meanwhile, PAMs from Anm and even more from AnmFe mice had lower FPN1 expression (Fig. 3g). As inability to increase FPN1 expression upon S.Tm infection is associated with impaired infection control and intracellular bacterial proliferation [47,48], these data indicate that PAMs of anaemic mice, even when supplemented with iron, cannot upregulate FPN1 to a sufficient extent to starve intracellular S.Tm from iron [40]. The results obtained thus far indicate that increased plasma iron and TSAT as well as the inability of macrophages to upregulate FPN1 correlate with adverse outcomes.

### 3.4 Effect of anaemia and iron status on the immune system

**Fig. 4:**
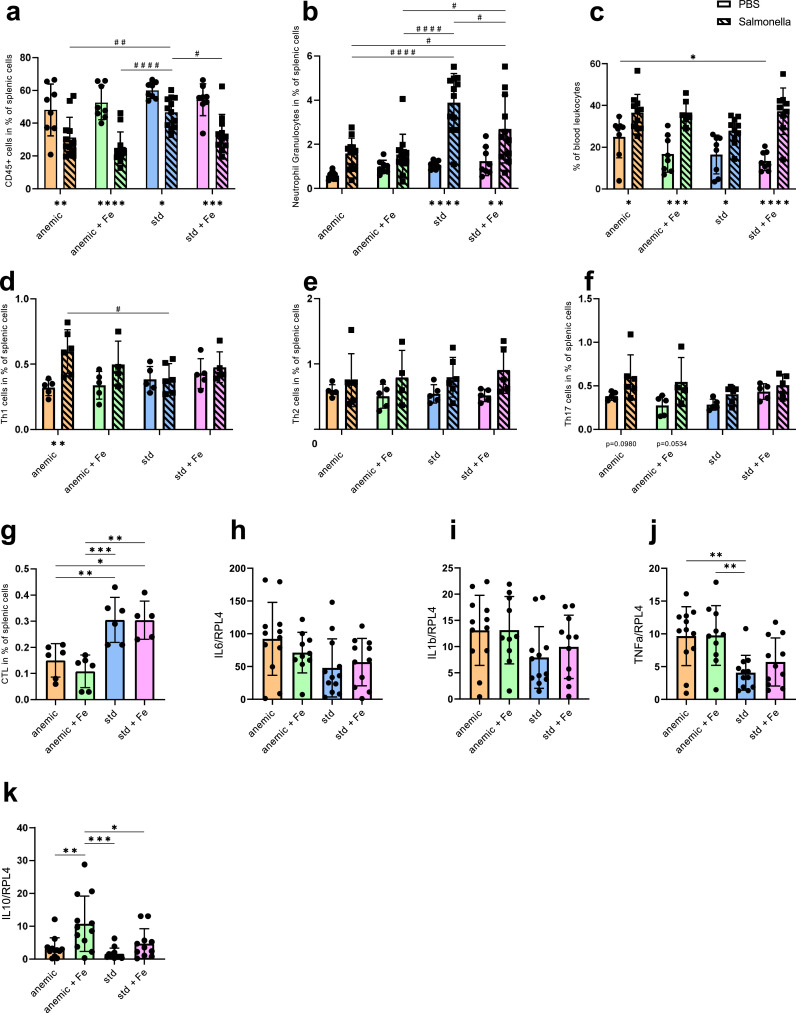
Effect of anaemia and iron status on the immune system. Mice were infected with 500 CFU of *Salmonella* Typhimurium (S.Tm) for 72 h. (a) Flow cytometry results showing the percentage of splenic leukocytes. Splenic leukocytes were defined as CD45+ cells. (b) Percentage of neutrophil granulocytes of all splenic cells. Neutrophil granulocytes were defined as CD45+, CD3-, CD19-, Ly6G+ cells. (c) Neutrophil granulocytes of the blood in percent of leukocytes. Blood count was performed using a Vet ABC. (d) Splenic Th1 cells in percent of all cells. Th1 cells are defined as CD45+, CD3+, CD8-, CD4+, IFNg+. (e) Splenic Th2 cells in percent of all cells. Th2 cells are defined as CD45+, CD3+, CD8-, CD4+, IL4+. (f) Splenic TH17 cells in percent of all cells. Th17 cells are defined as CD45+, CD3+, CD8-, CD4+, IL17A+. (g) Cytotoxic T cells were defined as CD45+, CD3+, CD19-, CD8a+, TNFa+, IFNg+. qPCR results of splenic (h) interleukin 6 (IL-6), (i) IL-1b, (j) TNFa and (k) gene expression of S.Tm infected mice. qPCR results were calculated using the delta-delta-ct method and *Rpl4* was used as a reference gene. Each dot/square indicates a single mouse. Results of (a–c) and (h–k) are from two identical performed experiments (PBS: *n* = 8, S.Tm: *n* = 10–12). Results of (d–g) and (i) are from a single experiment (PBS: *n* = 5, S.Tm: *n* = 4–6). One-way ANOVA with Tukey-corrected post-hoc t-tests for multiple comparison was applied for comparison between groups. Two-way ANOVA with Tukey-corrected post-hoc t-tests for multiple comparison was performed for the comparison of the different groups of infected and PBS treated mice. S.Tm infected mice are shown in bars with a diagonal line pattern and black squares for each mouse and PBS treated mice in blank bars with black circles for each mouse. Differences between infected and PBS treated are shown underneath the bars. When S.Tm and PBS treated mice are shown on the same graph significances between the S.Tm infected mice are shown using # and significances between PBS treated groups are shown using *. Data are presented as mean ± SD. *p < 0.05, **p < 0.01, ***p < 0.001, ****p < 0.0001, #*p* < 0.05, ##*p* < 0.01, ###*p* < 0.001, ####p<0.0001.

The observation that the anaemic mice had the lowest plasma iron levels but higher amounts of CFU than the Std mice, suggests an additional mechanism for the increased bacterial load in anaemic mice. As iron deficiency may impact on the functionality of the immune system by different pathways [23,51], we analyzed immune response pattern in the experimental groups. Thus, we used flow cytometry to study the role of various immune cells of the spleen. The gating strategy for all subtypes is depicted in the supplements (Suppl. Fig. 2a-b). Splenic leukocyte numbers (CD45 positive) did not show differences between all four groups at baseline. However, S.Tm infected mice that were initially on a low iron diet (Anm and AnmFe) and mice that received the standard diet followed by iron supplementation (StdFe) had lower numbers of splenic leukocytes compared to the mice that were kept on the standard iron diet (Std) (Fig. 4a). Neutrophil granulocyte (CD45+, CD3-, CD19-, Ly6G+) numbers in the spleen were highest in the infected Std group and lower in all other infected groups, with Anm and AnmFe mice having significantly reduced neutrophil numbers even when compared to StdFe mice (Fig. 4b). In contrast, no differences in the percentage of neutrophil granulocytes were found in the blood of infected mice (Fig. 4c).

We then looked for differences in T cell subsets in the spleen. CD4 positive T helper cells, specifically Th1 (CD45+, CD3+, CD8-, CD4+, IFNg+), Th2 (CD45+, CD3+, CD8-, CD4+, IL4+) and Th17 cells (CD45+, CD3+, CD8-, CD4+, IL17A+) (Fig. 4d–f) did not show any differences that could explain the phenotype. Interestingly, infected Anm mice had increased numbers of Th1 when compared to uninfected littermates or to infected Std mice (Fig. 4d).

The numbers of splenic CD8 positive cytotoxic T cells (CTL) (CD45+, CD3+, CD8+, TNFa+, IFNg+) were significantly reduced in infected Anm and AnmFe mice as compared to Std and StdFe mice (Fig. 4g).

In a next step, we had a look on various cytokines in the plasma using a multiplex ELISA. To solve the cause and effect question if increased cytokine levels result from increased bacterial load or from dysregulated iron status, we compared the data from S.Tm infected mice with data from mice on the same diets but injected with 2 mg/kg bodyweight of S.Tm lipopolysaccharide (LPS). The plasma levels of pro-inflammatory cytokines IL-6, TNFa, IL-1b and the anti-inflammatory cytokine IL-10 were increased in the groups with higher bacterial loads but did not show significant differences for all the groups stimulated with LPS (Suppl. Table. 1). IFNg was increased in S.Tm infected Anm mice, which is in agreement with higher numbers of Th1 cells (Suppl. Table. 1, Fig. 4d).

To study the alterations of cytokine levels in the different dietary groups at a local level, we analysed cytokine transcript levels in the spleen by qPCR. The expression levels of pro inflammatory cytokines *IL-6* and *IL-1b* did not show any differences between all four groups infected with S.Tm and LPS respectively (Fig. 4h,I, Suppl. Fig. 2c-d). *Tnfa* gene expression was higher in infected Anm and AnmFe mice in the spleen but did not show any differences in the LPS treated mice (Fig. 4j, Suppl. Fig. 2e). Interestingly, the transcription of anti-inflammatory cytokine *IL-10* was elevated in spleens of the AnmFe mice, when either being infected with S.Tm or treated with LPS (Fig. 4k, Suppl. Fig. 2f).

Taken together, these results show that, an anaemic state, even when supplemented with iron, followed by infection with S.Tm, impairs the immigration or proliferation of neutrophil granulocytes and cytotoxic T cells into the spleen, what is associated with a poorer survival of the AnmFe group. In addition, while increased IFN-g production may exert some protection in Anm mice as compared to AnmFe animals, the iron mediated increase of IL-10 may negatively impact on immune control of iron supplemented animals.

## 4 Discussion

Broad iron supplementation of children in low-income countries, regardless if they are iron deficient or not, is considered to be a helpful strategy to overcome negative side effects of iron deficiency, like growth and mental retardation [52]. In principle, iron supplementation of iron deficient children, infants and pregnant women is an important public health measure to prevent childhood morbidity, impaired cognitive development and school performance, what can occur early and might be irreversible [[53], [54], [55]].

However, since 2006 there is growing awareness of possible adverse effects of this treatment in areas with a high endemic burden of infection, as a randomized clinical trial in Pemba found increased mortality and morbidity in children which received iron supplementation [10]. This finding was confirmed by another clinical study demonstrating increased burden of infectious diseases in iron supplemented children [14]. The underlying mechanisms of these negative outcomes remain elusive and a detailed assessment to find and define patterns for individuals at risk has not been performed. Previous speculations favoured the notion that iron balanced children receiving additional iron supplementation might be at a higher risk for infections [10]. Numerous animal models together with human data have convincingly shown that high iron availability worsens the course of certain infections [[56], [57], [58], [59]].

Iron is an essential nutrient for both mammals and microbes [60]. It is therefore well established that control over iron homeostasis is of central importance in the host – pathogen interaction, where both opponents compete for iron [61]. Under those assumptions, it was surprising that the worst outcome of *Salmonella* infection was observed when iron was supplemented to iron deficient rather than iron balanced mice. This suggests that the ones expected to benefit the most from iron supplementation appear to be at the same time at the highest risk for severe infections.

The only study undertaken in this direction so far showed *ex vivo* that certain bacteria like *E.coli, Salmonella* and *Yersinia* grow better in sera of people which received one oral iron dose four hours before, in comparison to the sera before the supplementation [62]. Their results showed that a single dose of 2 mg/kg ferrous sulphate increased the TSAT from about 45 to 75%, and that the increased TSAT correlates with increased bacterial growth.

Based on the data presented herein, we hypothesize that the underlying mechanism of the observed phenomenon is multifactorial and propose a “two hit” model including iron availability and immune cell function.

The first and probably main hit can be attributed to the high iron availability in AnmFe mice. Iron supplementation resulted in the expansion of iron pools in organs but also in increased circulating iron levels as reflected by higher TSAT and the emerge of NTBI, specifically in AnmFe mice. Bacteria, like *Salmonella*, may thus benefit from the NTBI before they enter the cells, and further from increased iron availability in their specific organ environment [63]. This is supported by the finding that *sodB*, whose expression is affected by intracellular iron levels [41], is upregulated in the AnmFe group, indicating that *Salmonella* readily incorporate the available iron. Importantly, *Salmonella* that are lacking two major iron uptake systems (*∆entC ∆feo*) and one Mn^2+^ / Fe^2+^ transporter (*∆sitABCD)* did not show differences in proliferation kinetics between the four animal groups. This points to the pivotal importance of bacterial iron availability for bacterial growth in iron supplemented animals.

A further aggravating factor to the poor infection control in AnmFe mice is likewise originating from altered expression of the iron exporter FPN1. While anaemic mice are fully depleted in their iron stores, macrophages and other body cells might try to acquire as much iron as possible upon iron availability by iron supplementation and therefore the FPN1 level remain low even after iron supplementation. This in turn results in easy accessible iron source for invading *Salmonella* and consequent promotion of their growth [40].

Of note, when comparing hepcidin levels and macrophage FPN1 expression between Std and Anm groups, it appears that FPN1 is regulated not only by circulating hepcidin levels, but additionally via translational regulation involving iron regulatory proteins or pathogen mediated regulatory circuits [33,64,65]. Further, FPN1 regulation underlies a dynamic process depending on the time point of investigation and the mode of *Salmonella* infection. Different expression pattern have been described in various tissues and cells [40,[66], [67], [68], [69], [70]].

In addition, StdFe mice have higher iron levels than AnmFe in the spleen, what may be linked to the fact that anaemic mice may shift iron directly to the bone marrow for erythropoiesis. This would be in line with almost normalized Hb levels of uninfected AnmFe mice (Fig. 1b) and a smaller increase of Hb in infected AnmFe mice due to inflammatory driven iron retention in the spleen (Fig. 2e, Suppl. Fig. 1b).

FT may be a poor indicator of stored iron in the setting of *Salmonella* infections as its protein levels are not paralleled by tissue iron quantification and Prussian blue staining (Fig. 2g,h). Therefore, increased FT may reflect ongoing inflammation as many cytokines induce expression of H- and L-ferritin, the later lacking ferroxidase activity [71].

The second hit of our theory is the negative effect of anaemia on various immune cells, shown in the poorer outcome of anaemic mice as compared to Std and StdFe littermates. Neutrophil granulocytes play an important role in the defence against bacteria [[72], [73], [74]], but due to anaemia their accumulation in affected tissues such as the spleen is impaired. This could be due to e.g. splenic extra-medullary myelopoiesis [75], reduced splenic pools of neutrophils [76] or impaired or diminished immigration of neutrophil granulocytes into the spleen.

Additionally, iron overload can lead to neutrophil granulocytes dysfunctions like impaired phagocytosis, or impaired generation of superoxide anion (O2-) [77], what could play a role as well in our model, since iron supplemented groups (AnmFe, StdFe) have higher bacterial loads than not-supplemented ones.

Of note, we also found reduced numbers of cytotoxic CD8+ T cells in the spleen of infected Anm and AnmFe animals, resulting in an impaired immune control as CTL have been shown to exert important functions in protective adaptive immunity to *Salmonella* Typhimurium infection in mice immunity [78,79]. This agrees with *in vitro* data showing that CD8+ cell numbers are affected by genetic alterations of iron homeostasis [80].

When studying other T cell subsets, we only found a slight increase in Th1 counts and associated formation of IFNg in anaemic mice. This would be in line with effects of iron restriction on IFNg formation and Th1 expression and improved immune control of infection [59,81,82]. However, it appears that this potentially favourable immune response condition is not able to compensate for deficiency in other immune effector pathways in anaemic mice. Our cytokine analysis revealed an increased expression of the macrophage de-activating cytokine IL-10 in the AnmFe, but also a slight increase in StdFe mice, what can further favour bacterial growth by weakening anti-microbial host immune defences of macrophages [83]. As the same regulation was seen in LPS treated mice, it is likewise caused by iron accumulation, although the underlying mechanisms of IL-10 induction in that setting have not been fully elucidated thus far [84]. This would be in accordance with the observation that anaemic mice have a poorer outcome because of an impaired immune function resulting from iron restriction to immune cells including T cells. Iron supplementation to those animals further aggravates this situation by stimulating anti-microbial immune effector pathways (IL-10) and increasing the accessibility of iron to bacteria. Further studies involving IL-10 knockout mice in our experimental setting may contribute to a deeper understanding of the interaction between IL-10, anaemia and iron supplementation for infection control, given the known associations between IL-10 and iron dysregulation in inflammation [84,85].

In summary, our results indicate that iron supplementation of anaemic mice worsens the course of bacterial infection based on “two hits”. First, due to iron supplementation, bacteria enter an iron rich environment which enhances their proliferation. Second, anaemia and/or severe iron deficiency negatively affects various immune cell functions as they don't proliferate in comparison to iron balanced mice (Std), thereby negatively affecting anti-microbial immune responses. We could show that each hit on its own resulted in an increased bacterial load and that both hits together have an additive effect.

In terms of iron supplementation strategies to children in areas with a high endemic burden of infection, our data suggests an increased risk of adverse outcomes from infection specifically among anaemic individuals receiving iron supplementation. This would led one hypothesize that iron supplementation to anaemia children in areas with high endemic burden of infections or chronic infections should be performed cautiously, and that short course anti-microbial or anti-parasitic treatments before iron supplementation may be indicated. To see whether our findings also hold true in humans, prospective randomized trials are mandatory and urgently needed. Specifically, accessing pre-therapy iron homeostasis, haemoglobin levels and immune function at baseline, in addition to the quantification of infection risk or an adverse outcome of the patients during follow up, would be of major importance.

## Contributors

AH, DH and GW planed and designed the project. AH, DH, LVS, PT, MS, LR, RH,VP and PM performed experiments. AH, DH, PT and MS analysed data. AH and DH performed the statistical analysis. AH and DH did the visualization of the data. AH, DH, MN and GW prepared and created the initial draft. LVS, PT, LR, VP and MN were included in the critical review and writing of the manuscript. GW was responsible for supervision, conceptualization, and funding acquisition. All authors read and approved the final manuscript.

## Data sharing statement

The data that support the findings of this study are available from the corresponding author GW upon reasonable request.

## Declaration of Competing Interest

The authors declare that they have no conflict of interest.

## Appendix Supplementary materials

*Suppl. Fig. 1.* (a) Haematocrit of PBS treated and infected animals receiving the different iron diets. (b) Absolute amount splenic iron of infected animals receiving the different iron diets. (c-h) Bacterial gene expression of infected spleens from different fed mice (c) *invF,* (d) *sicA*, (e) *ssaG*, (f) *sseA*, (g) *sitB* and (h) the iron homeostasis regulator fur (one outlier in the group of Anm, AnmFe and Std respectively). (i) Transferrin Elisa from plasma of infected and uninfected mice receiving the different diets. Densitometry quantification of splenic (j) transferrin (TFR) and (k) ferritin (FT) from western blot results. Each dot indicates a single mouse. Results of (a-d) and (g) are from two identical performed experiments (*n* =10–12). Results of (e,f) and (h–k) are from a single experiment (*n* = 3–6). One-way ANOVA was applied for comparison between groups. Two-way ANOVA with Tukey-corrected post-hoc t-tests for multiple comparison was performed for the comparison of the different groups of infected and PBS treated mice. S.Tm infected mice are shown in bars with a diagonal line pattern and black squares for each mouse and PBS treated mice in blank bars with black circles for each mouse. When S.Tm and PBS treated mice are shown on the same graph significances between the S.Tm infected mice are shown using # and significances between PBS treated groups are shown using *. Differences between infected and PBS treated are shown underneath the bars. Data are presented as mean ± SD. **p* < 0.05, ***p* < 0.01, ****p* < 0.001, *****p* < 0.0001, #*p* < 0.05, ##*p* < 0.01, ###*p* < 0.001, ####*p* < 0.0001. Image, application 1

Image, application 2

Image, application 3
