# Supplementary material for: Baseline iron status and presence of anaemia determine the course of systemic Salmonella infection following oral iron supplementation in mice
Source: eBioMedicine. 2021 Sep 3;71:103568. doi: 10.1016/j.ebiom.2021.103568 (PMC8426537; doi:10.1016/j.ebiom.2021.103568)
Supplement: Supplementary file 2 [file mmc2.pdf]

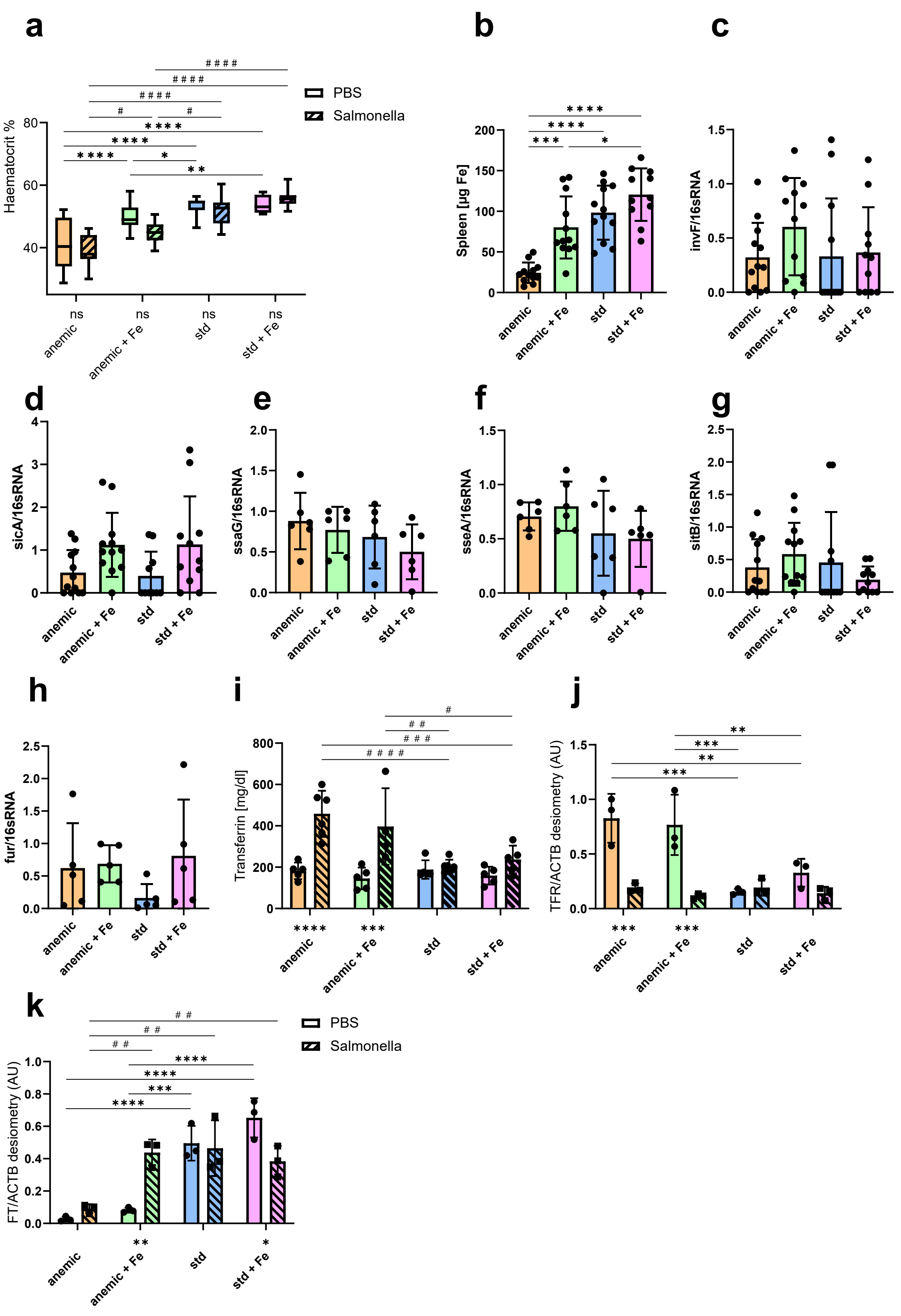

a
b
c
Haematocrit %
PBS
Salmonella
ns
anemic
anemic + Fe
std
std + Fe
Spleen [µg Fe]
invF/16sRNA
d
e
f
g
sicA/16sRNA
ssaG/16sRNA
sseA/16sRNA
sitB/16sRNA
h
i
j
fur/16sRNA
Transferrin [mg/dl]
TFR/ACTB desiometry (AU)
k
FT/ACTB desiometry (AU)

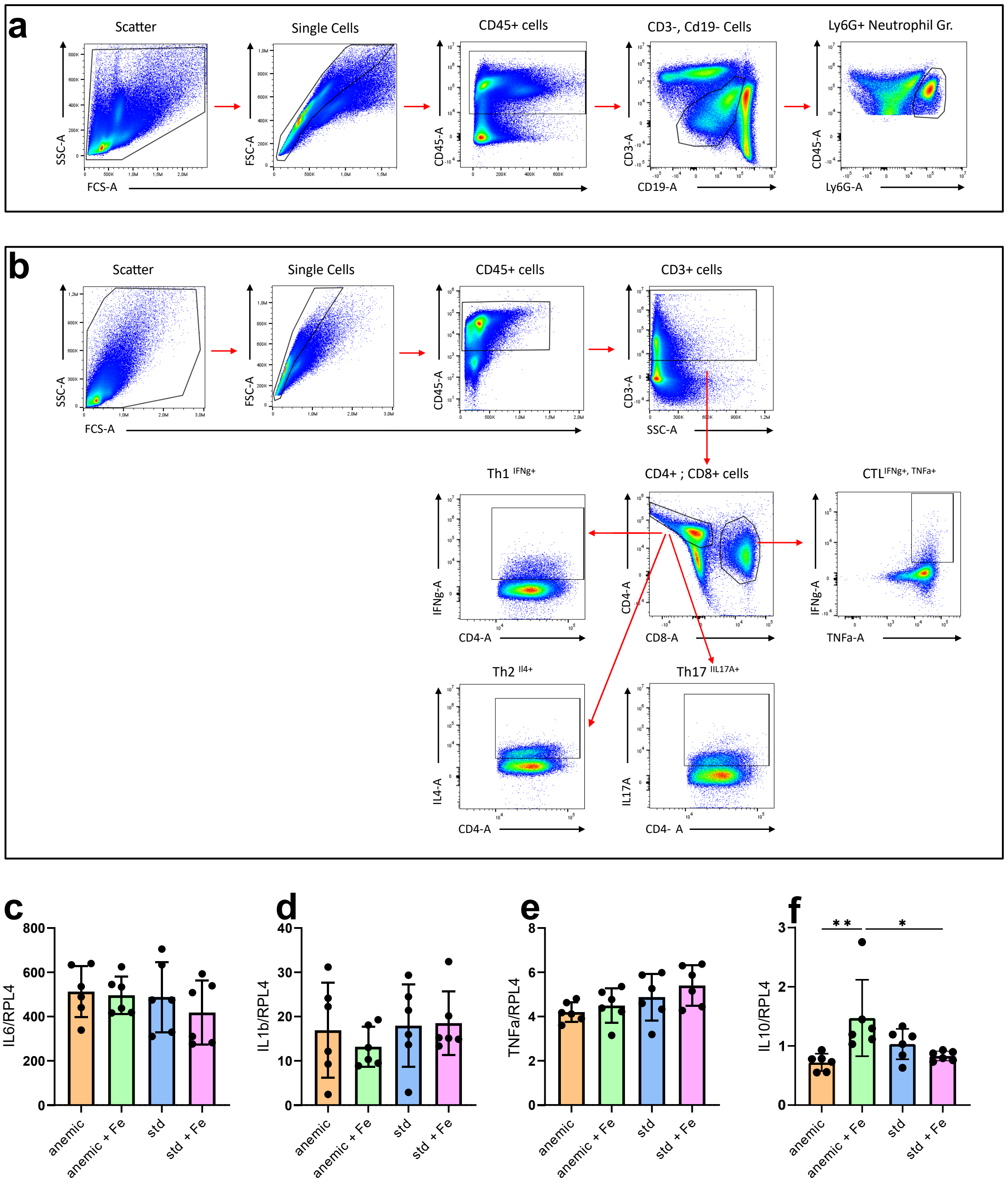

a
Scatter
Single Cells
CD45+ cells
CD3-, Cd19- Cells
Ly6G+ Neutrophil Gr.
SSC-A
FCS-A
FSC-A
CD45-A
CD3-A
CD19-A
Ly6G-A
b
Scatter
Single Cells
CD45+ cells
CD3+ cells
SSC-A
FCS-A
FSC-A
CD45-A
CD3-A
Th1 IFNg+
CD4+ ; CD8+ cells
CTL IFNg+, TNFa+
IFNg-A
CD4-A
CD8-A
TNFa-A
Th2 Il4+
Th17 IIL17A+
IL4-A
IL17A
CD4- A
c
IL6/RPL4
d
IL1b/RPL4
e
TNFa/RPL4
f
IL10/RPL4
anemic
anemic + Fe
std
std + Fe
**
*

		INF-gamma (pg/ml)	IL-1beta (pg/ml)	IL-12p70 (pg/ml)	IL-2 (pg/ml)	IL-6 (pg/ml)	TNF-alpha (pg/ml)	IL-10 (pg/ml)
PBS	anemic	0,0 ± 0,0	0,0 ± 0,0	0,0 ± 0,0	0,0 ± 0,0	0,0 ± 0,0	1,4 ± 1,1	0,0 ± 0,0
	anemic + Fe	0,0 ± 0,0	0,0 ± 0,0	0,0 ± 0,1	0,5 ± 0,7	0,0 ± 0,0	1,7 ± 0,5	0,0 ± 0,0
	std	0,0 ± 0,0	0,0 ± 0,0	0,1 ± 0,1	0,0 ± 0,0	0,0 ± 0,0	1,0 ± 0,8	0,0 ± 0,0
	std + Fe	0,0 ± 0,0	0,0 ± 0,0	0,0 ± 0,0	0,2 ± 0,4	0,0 ± 0,0	1,1 ± 1,1	0,0 ± 0,0
Salmonella	anemic	1292,2 ± 1020,4	1,2 ± 3,9	15,1 ± 9,8	1,7 ± 0,9	962,0 ± 968,6	168,4 ± 103,2	4,9 ± 14,9
	anemic + Fe	629,7 ± 402,9 ** * *	5,2 ± 8,5	7,0 ± 4,4	1,7 ± 1,0	1430,4 ± 964,4 **	279,9 ± 124,4 **	27,4 ± 48,0
	std	648,2 ± 428,1	0,0 ± 0,0	13,2 ± 10,3	1,4 ± 1,0	193,5 ± 135,7	67,2 ± 69,0	0,0 ± 0,0
	std + Fe	659,8 ± 368,4	4,2 ± 9,3	15,1 ± 12,9	2,2 ± 1,1	761,9 ± 726,0	222,6 ± 160,0 *	30,9 ± 73,8
LPS	anemic	615,5 ± 319,0	17,7 ± 6,9	6,6 ± 0,6	2,3 ± 0,3	33456,4 ± 16809,0	207,1 ± 82,4	42,8 ± 12,6
	anemic + Fe	441,5 ± 352,3	16,4 ± 10,3	5,5 ± 1,2	2,0 ± 0,2	18601,7 ± 8798,6	216,9 ± 107,6	41,4 ± 31,0
	std	367,0 ± 426,7	16,4 ± 13,3	4,7 ± 1,3	1,6 ± 0,9	24341,6 ± 33296,6	183,5 ± 44,5	23,4 ± 32,1
	std + Fe	451,2 ± 432,8	11,5 ± 7,8	3,9 ± 0,6	1,6 ± 0,9	15864,5 ± 12694,3	204,1 ± 28,9	18,7 ± 23,8
