# Supplementary material for: Baseline iron status and presence of anaemia determine the course of systemic Salmonella infection following oral iron supplementation in mice
Source: eBioMedicine. 2021 Sep 3;71:103568. doi: 10.1016/j.ebiom.2021.103568 (PMC8426537; doi:10.1016/j.ebiom.2021.103568)

Original Western Blots from **Figure 3 D**. Used marker was aBio Rad Precision Plus Protein (Cat# 1610373)

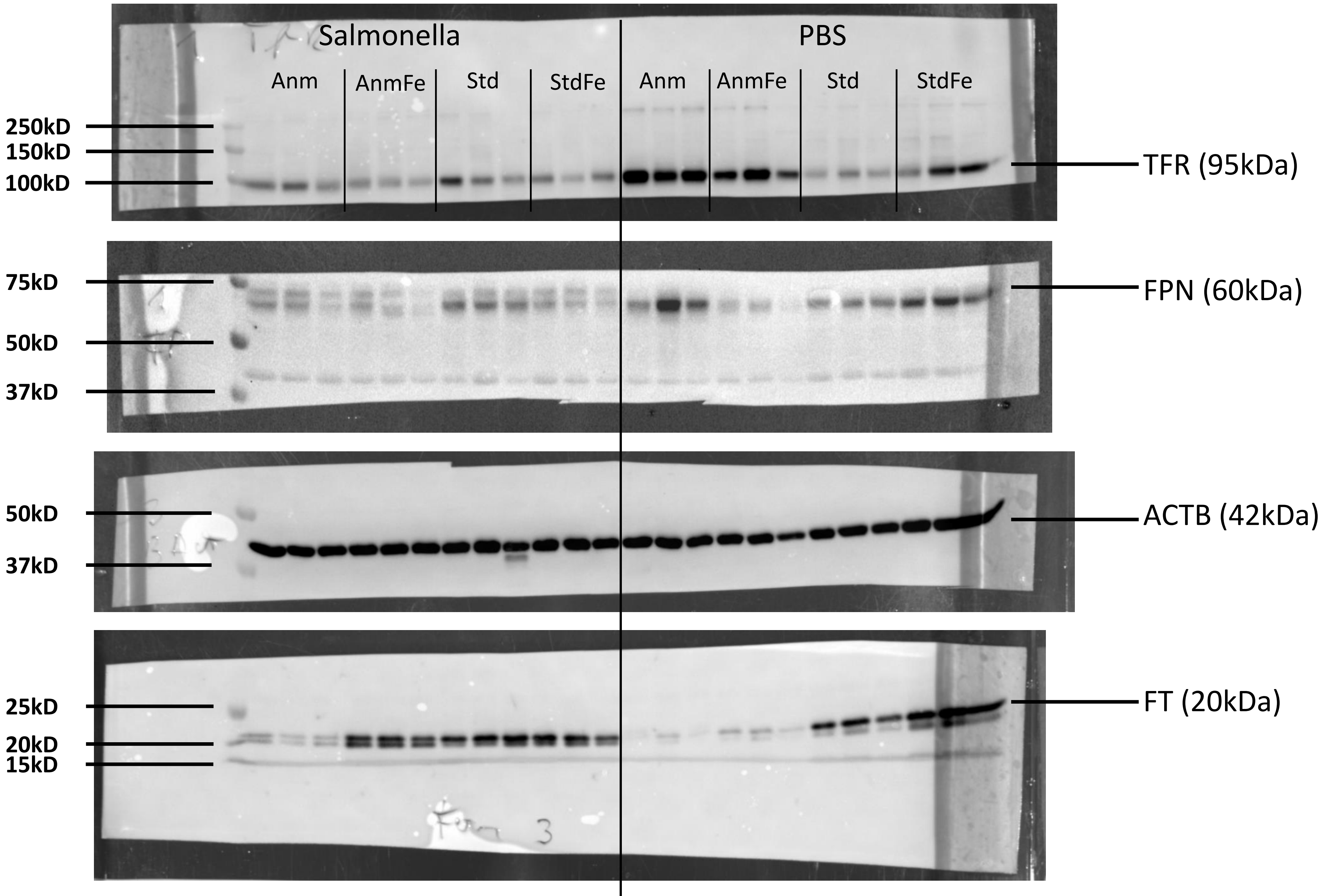

Original Western Blots from **Figure 3 G**. Used marker was aBio Rad Precision Plus Protein (Cat# 1610373).
X: samples that did not make it to the results (light iron deficiency).

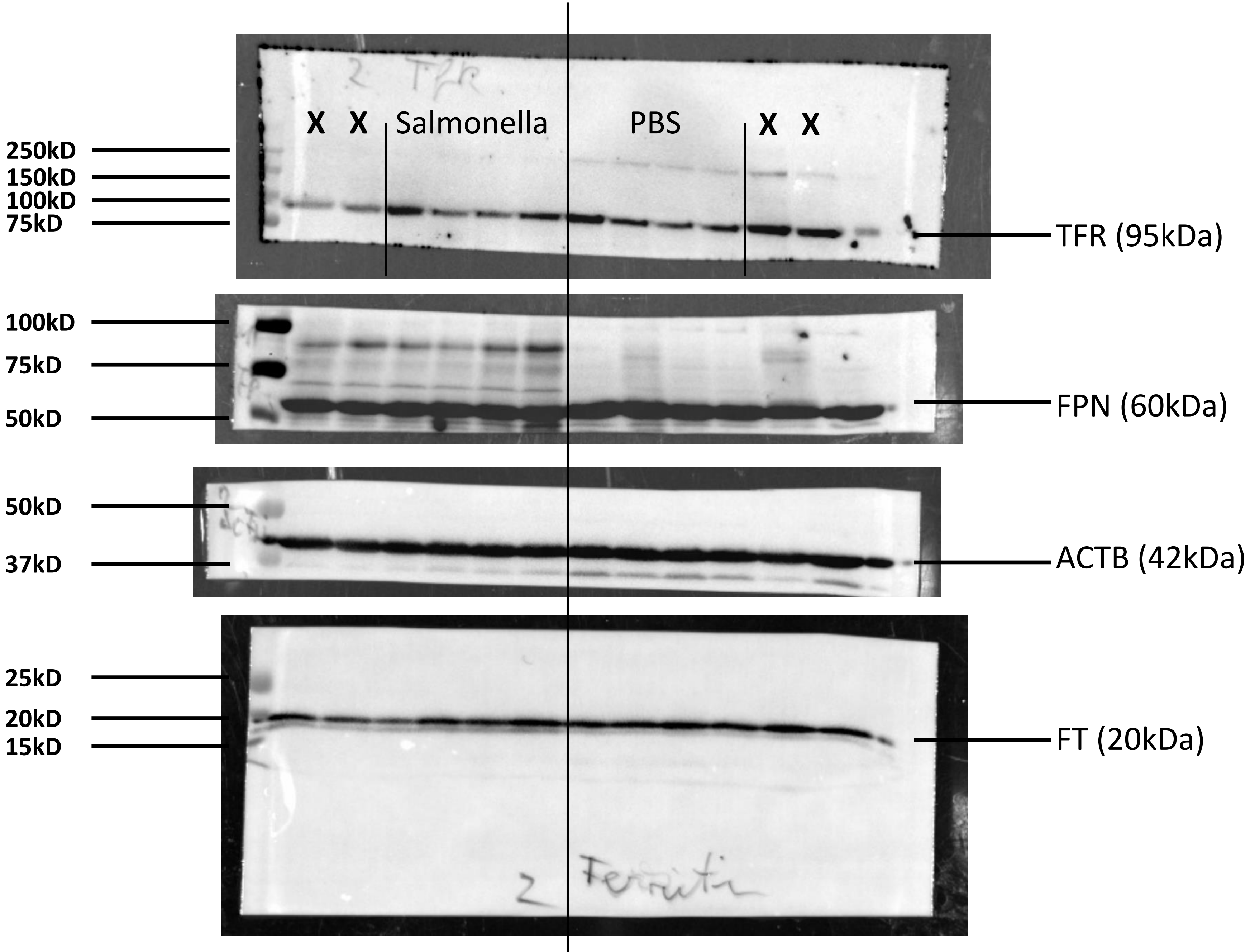

Supplement: Supplementary file 3 [file mmc3.pdf]
